# Smoking and Hypertriglyceridemia Predict ST-Segment Elevation Myocardial Infarction in Kosovo Patients with Acute Myocardial Infarction

**DOI:** 10.3390/clinpract14030091

**Published:** 2024-06-17

**Authors:** Afrim Poniku, Arlind Batalli, Dua Shita, Zarife Rexhaj, Arlind Ferati, Rita Leka, Artan Bajraktari, Genc Abdyli, Edmond Haliti, Pranvera Ibrahimi, Rona Karahoda, Shpend Elezi, Faik Shatri, Ibadete Bytyçi, Michael Henein, Gani Bajraktari

**Affiliations:** 1 Medical Faculty, University of Prishtina, 10000 Prishtina, Kosovo; afrim.poniku@uni-pr.edu (A.P.); dua.shita@student.uni-pr.edu (D.S.); gencabdyli@yahoo.com (G.A.); edihal@yahoo.com (E.H.); gani.bajraktari@uni-pr.edu (G.B.); 2 Clinic of Cardiology, University Clinical Centre of Kosova, 10000 Prishtina, Kosovo; dr.zariferexhaj@gmail.com (Z.R.); arlind1ferati@gmail.com (A.F.); ritaleka11@gmail.com (R.L.); artanbajraktari@hotmail.com (A.B.); pranvera_i86@hotmail.com (P.I.); selezi@hotmail.com (S.E.); faikshatri@hotmail.com (F.S.); i.bytyci@hotmail.com (I.B.); 3 Institute of Public Health and Clinical Medicine, Umeå University, 90187 Umeå, Sweden; michael.henein@umu.se; 4 Research Unit, Heimerer College, 10000 Prishtina, Kosovo; rona.karahoda@kolegji-heimerer.eu

**Keywords:** myocardial infarction, risk factors, smoking, diabetes, arterial hypertension, age, gender

## Abstract

Background: Myocardial infarction (MI), presented as ST-segment elevation MI (STEMI) and non-ST-segment elevation MI (NSTEMI), is influenced by atherosclerosis risk factors. Aim: The aim of this study was to assess the patterns of presentation of patients with acute MI in Kosovo. Methods: This was a cross-sectional study conducted at the University Clinical Center of Kosovo, which included all patients hospitalized with acute MI over a period of 7 years. Results: Among the 7353 patients admitted with acute MI (age 63 ± 12 years, 29% female), 59.4% had STEMI and 40.6% had NSTEMI. The patients with NSTEMI patients less (48.3% vs. 54%, *p* < 0.001), but more of them had diabetes (37.8% vs. 33.6%, *p* < 0.001), hypertension (69.6% vs. 63%, *p* < 0.001), frequently had a family history of coronary artery disease (CAD) (40% vs. 38%, *p* = 0.009), and had more females compared to the patients with STEMI (32% vs. 27%, *p* < 0.001). The patients with NSTEMI underwent less primary percutaneous interventions compared with the patients with STEMI (43.6% vs. 55.2%, *p* < 0.001). Smoking [1.277 (1.117–1.459), *p* ˂ 0.001] and high triglycerides [0.791 (0.714–0.878), *p* = 0.02] were independent predictors of STEMI. Conclusions: In Kosovo, patients with STEMI are more common than those with NSTEMI, and they were mostly males and more likely to have diabetes, hypertension, and a family history of CAD compared to those with NSTEMI. Smoking and high triglycerides proved to be the strongest predictors of acute STEMI in Kosovo, thus highlighting the urgent need for optimum atherosclerosis risk control and education strategies.

## 1. Introduction

The presence of atherosclerotic plaques in the coronary arterial walls can be asymptomatic for years and even decades in many patients [1]. In some patients, the progression of atherosclerotic plaques is slow, and they gradually become symptomatic, presenting with stable angina with exertion, but others may present with acute myocardial infarction (MI) [2]. In recent decades, the epidemiological patterns of coronary artery disease (CAD) and acute MI have changed worldwide [3], with some European countries showing a significant fall in the incidence of acute CAD cases [4,5], although this pattern is not consistent across the whole of Europe with the same extent. Despite that, CAD remains the main global cause of death, particularly in low- and middle-income countries [6,7]. Moreover, the epidemiology and risk factors for CAD differ, being impacted by differences in geographic, socio-economic, and environmental conditions [8,9,10,11,12,13,14]. The highest increases in the incidence of cardiac events have been seen in Latin America and the Middle East, with significant regional variations [15]. In addition, the mortality from CAD increased dramatically in recent decades in developing countries (China, India, Latin America, etc.) [15]. The relationship between the increased occurrence of CAD and traditional risk factors seems to be different in different geographical areas. The high occurrence of CAD in China was associated with conventional risk factors, but this was not the case in India [16,17]. In particular, smoking and dyslipidemia do not consistently hold the same risk impact in all countries. 

Recent decades have witnessed a significant improvement in the diagnosis and treatment of acute MI, emphasizing immediate diagnosis and risk stratification, as well as introducing interventional revascularization treatment in all patients with acute MI [18,19]. This resulted in a significant decline in the incidence of ST-segment elevation MI (STEMI) over the years, whereas that of non-ST-segment elevation MI (NSTEMI) gradually increased, most likely due to the advent of high-sensitivity biomarkers [20,21,22]. The epidemiology and risk factors of CAD and acute MI in the general population and in patients with known CAD in the Republic of Kosovo are still not well determined [23]. Therefore, the objective of this study was to assess the occurrence, clinical characteristics, and impact of risk factors in patients with different types of acute MI in Kosovo. 

## 2. Materials and Methods

This study was conducted at the Clinic of Cardiology of the University Clinical Center of Kosovo, Prishtina using a cross-sectional descriptive analysis. All patients hospitalized with acute MI (a total of 7353 patients) over a period of 7 years (from 1 January 2014 to 31 December 2020) were included in this study. After receiving approval from the Ethical Council of the Kosovo Doctors Chamber, data were collected by a researcher who administered a structured questionnaire to all patients with acute MI presented during the designated study period. The demographic data from the patients’ records, including age, gender, risk factors (smoking, arterial hypertension, diabetes, dyslipidemia, and family history of CAD), clinical data (cardiogenic shock, duration of hospitalization, and outcome), biochemical data (glycemia at admission, cholesterol, triglycerides, urea, creatinine, hemoglobin, and troponin), electrocardiogram, medications, and data from an echocardiographic examination at admission were all collected. In addition, conventional coronary angiographic data and the results of the interventional treatment were collected. Acute MI was diagnosed based on the electrocardiogram and conventional raised levels of myocardial biomarkers. The patients were divided into two groups based on the ST-segment elevation at admission: (1) non-ST-segment elevation myocardial infarction (NSTEMI) and (2) ST-segment elevation myocardial infarction (STEMI). 

We compared the data of our patients with acute MI from Kosovo with those from Northern (Finland) and Southern (Greece) Europe [24].

### Statistical Analysis

The patients’ clinical characteristics were collected retrospectively from the medical records, which then underwent several thorough statistical analyses. Continuous variables are presented as mean ± SD, and categorical variables are presented as frequencies and percentages. A logistic regression analysis was performed to determine the predictors of STEMI in the study’s patients. Significance was determined based on a *p*-value < 0.05. Statistical analyses were performed using the IBM SPSS Statistics for Windows Operating System software, version 24.0 (IBM Corp., Armonk, NY, USA). Wald test for predictors was used to identify predictors of myocardial infarction. Kosovo patients with acute MI were compared with the respective patients from Northern (Finland) and Southern (Greece) Europe.

## 3. Results

### 3.1. General Data of Patients with AMI

In this study, we included 7353 patients admitted with acute MI (mean age 63 ± 12 years, 29% female) during a seven-year period (2014–2022). According to the final discharge diagnosis, 4366 (59.4%) patients were identified with STEMI, and 2987 (40.6%) were identified with NSTEMI. Among all patients admitted with acute MI, 1188 (16%) were transferred to another primary PCI hospital, whereas 84% remained for treatment at our center. 

### 3.2. Patients with STEMI Versus NSTEMI

The patients’ ages were not different between the two groups (63 ± 11 vs. 64 ± 12 years, *p* = 0.077), but the NSTEMI cohort had fewer smokers (48.3% vs. 54%, *p* < 0.001), more patients with diabetes (37.8% vs. 33.6%, *p* < 0.001), more patients with hypertension (69.6% vs. 63%, *p* < 0.001), more patients with a family history of CAD (40% vs. 38%, *p* = 0.009), and more females (32% vs. 27%, *p* < 0.001) compared to the STEMI cohort (Table 1). At admission, patients with NSTEMI had lower glucose (9.2 ± 5 vs. 9.8 ± 6 mmol/L, *p* < 0.001) and higher triglyceride (2.0 ± 1.2 vs. 1.88 ± 1.3 mmol/L, *p* = 0.001) levels compared with the patients with STEMI, whereas the cholesterol (4.8 ± 1.6 vs. 4.5 ± 1.5 mmol/L, *p* = 0.939), urea (9.2 ± 6 vs. 9.1 ± 6 mg/dL, *p* = 0.652), creatinine (118 ± 74 vs. 117 ± 73 μmol/L, *p* = 0.632), and hemoglobin (13.7 ± 3.4 vs. 13.7 ± 3.0 g/dL, *p* = 0.942) levels did not differ between the two cohorts (Table 1). Also, at admission, the patients with NSTEMI had less atrial fibrillation (3.9% vs. 5%, *p* = 0.035), less left bundle branch block (2.6% vs. 5.3%, *p* < 0.001), higher left ventricular ejection fraction (51.6 ± 9% vs. 49.8 ± 9%, *p* < 0.001), and less cardiogenic shock (2.1% vs. 4.9%, *p* < 0.001) compared to the patients with STEMI (Table 1). 

Almost two-thirds of the patients had significant CA stenosis, but there was no difference between the two cohorts with respect to the presence of significant CAD on diagnostic coronary angiography (66.8% vs. 67.8%, *p* = 0.396). The patients with NSTEMI underwent less primary percutaneous interventions (PCIs) compared with the patients with STEMI (43.6% vs. 55.2%, *p* < 0.001) (Table 2). 

### 3.3. Risk Factors and Predictors of STEMI

Smoking [1.277 (1.117–1.459), *p* ˂ 0.001] and a high triglyceride level [0.791 (0.714–0.878), *p* = 0.02] were independent predictors of STEMI (Table 3). 

### 3.4. Age and Gender in Patients with Acute AMI

**Figure 1:**
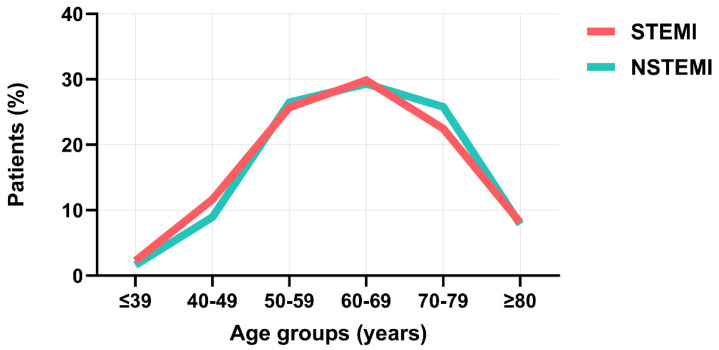
Age distribution of sample in patients with STEMI and NSTEMI.

**Figure 2:**
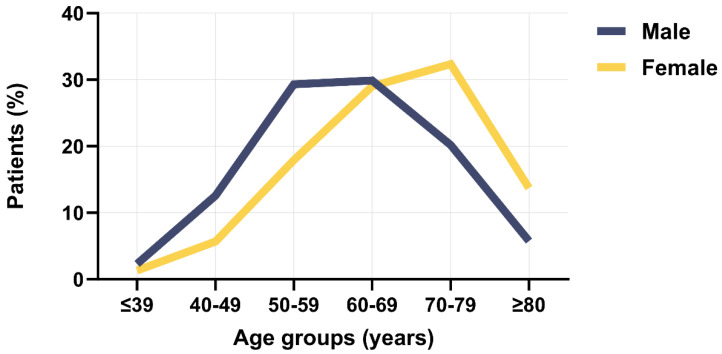
Age distribution of sample in male and female patients with acute myocardial infarction.

The highest percentage of patients with acute MI belonged to the 60–69-year age group (29.6%), followed by the 50–59-year age group (26%) (Figure 1). These percentages were not influenced by the type of acute MI the patients had, namely NSTEMI or STEMI (Figure 1). The highest percentage of female patients with acute MI belonged to the 70–79-year age group (32.3%), followed by the 60–69-year age group (29%), whereas the highest percentage of male patients was among the 60–69-year age group (29.9%), followed by the 50–59-year age group (29.3%) (Figure 2). Thus, there was a 10-year difference in the gender-determined occurrence of acute MI. 

#### Comparison between Acute MI Patients in Kosovo and Northern and Southern Europe

**Figure 3:**
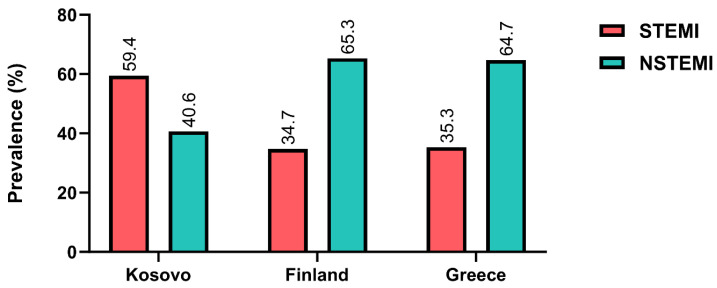
Prevalence of STEMI and NSTEMI in patients with acute myocardial infarction in Kosovo, Finland, and Greece.

**Figure 4:**
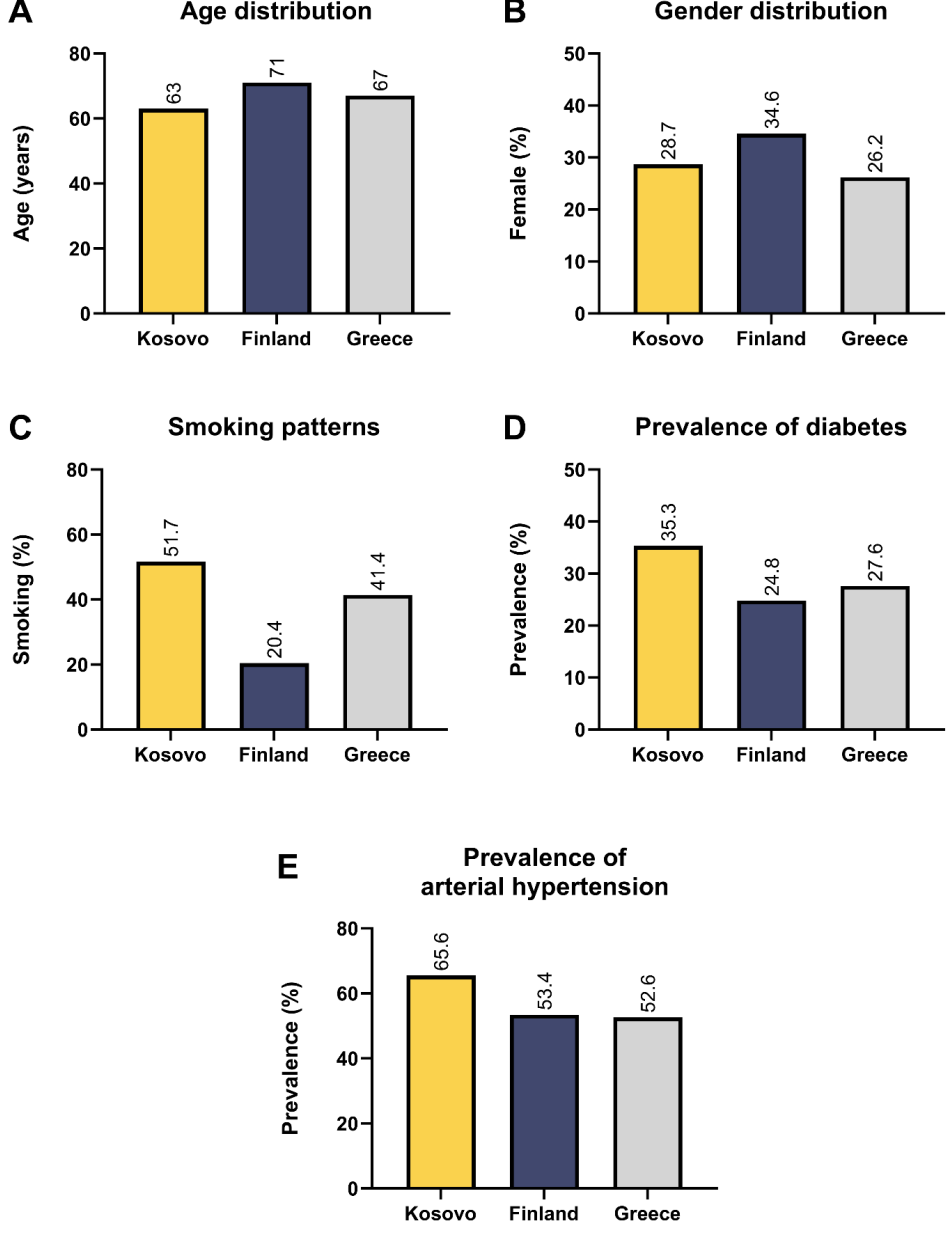
Age (**A**) and gender (**B**) differences and smoking (**C**), diabetes (**D**), and arterial hypertension (**E**) prevalence rates of patients with acute myocardial infarction between Kosovo, Finland, and Greece.

STEMI occurred significantly more frequently in patients with acute MI in Kosovo compared to those in Finland and Greece (Table 4 and Figure 3), but there was no difference between the latter two countries. Kosovo patients with acute MI were younger than those in Finland and Greece (*p* < 0.001, Figure 4A). The percentage of females admitted with acute MI in Kosovo was lower than those admitted in Finland and higher than those admitted in Greece (*p* < 0.001, Figure 4B). The prevalence rates of smoking, diabetes mellitus, and arterial hypertension were higher in Kosovo compared to Finland and Greece (*p* < 0.001, Figure 4C–E). While the prevalence of diabetes and hypertension did not differ between Finland and Greece, the prevalence of smoking was higher in Greece compared to Finland (Table 4, Figure 4C–E). 

## 4. Discussion

The aim of this observational study was to provide a real contemporary descriptive picture of patients presenting with acute MI who were admitted to the University Clinical Centre of Kosova, the only tertiary healthcare center in Kosovo, during a 7-year period and to identify the areas of the overall management strategy of such patients that require improvement. The findings of this study also reflect the pattern of atherosclerosis risk factors these patients had and its relationship with acute MI as well as the extent of differences of such pattern in comparison with other European countries. This study represents the largest real population survey of almost all patients referred with acute MI for optimum treatment, from all regional hospitals of Kosovo to our heart center; hence, the patient recruitment process should be described as consecutive.

Findings: Our data analysis shows that in Kosovo, females suffered less MIs than males. The majority of cases presenting with acute MI had STEMI and less commonly had diabetes, arterial hypertension, and a family history of CAD, but they smoked more compared to those presenting with NSTEMI. Also, Kosovo patients with STEMI had a higher incidence of electric disease in the form of atrial fibrillation, left bundle branch block, and lower left ventricular ejection fraction and more cardiogenic shock compared to the patients with NSTEMI. In addition, females were more prevalent among the NSTEMI group compared to the STEMI group. Moreover, the highest percentage of patients with acute MI belonged to the 60–69-year age group, with the highest percentage of females being in the 70–79-year age group. Finally, Kosovo patients admitted with acute MI had specific characteristics when compared with other European countries irrespective of their geographical location, namely north (Finland) or south (Greece). The majority of Kosovo patients presented with STEMI compared with the rest of Europe, who presented with NSTEMI. The Kosovo patients with acute MI were younger than the Finland patients by an average of 8 years and younger by 4 years compared to the Greek patients. Over 50% of the Kosovo patients smoked compared to only 20% of Finnish and 40% of Greek patients. The prevalence rates of diabetes and hypertension were also significantly higher in the Kosovo patients compared to the other two European cohorts. 

Data interpretation: Our analysis shows that smoking and a raised triglyceride level are the strongest predictors of STEMI. This reflects a cultural issue when compared with predictors in other countries, where hypercholesterolemia is a well-established factor [25,26]. In the absence of a significant role of diabetes in predicting acute MI in our cohort, the accumulative risk of smoking and hypertriglyceridemia could even explain the difference in MI presentation being mostly STEMI compared with that in other European countries, in which smoking and hypertensin are well controlled and most MI presentations are in the form of NSTEMI. This difference also reflects better established health services and patient education programs in the other European countries, particularly the northern ones, where smoking prevalence is significantly lower than that in the Mediterranean counterparts, as our analysis showed. The above interpretation could also explain the high prevalence of females admitted with acute MI compared with Greece, who are expected to lag behind males in developing atherosclerosis by approximately 10 years [27]. Furthermore, the above proposed interpretation likely explains the significant young age at admission that we found in our Kosovo patients compared with Finland and Greece. People usually start smoking early in life; hence, this factor contributes to the significant young age at presentation with STEMI, which our analysis showed. In addition, bad eating habits and a lack of exercise, starting early in life, are likely explanations for the significant predictive role of raised blood triglyceride levels as a predictor of STEMI. Smoking is a well-established risk for atherosclerosis and cancer [28,29]. On the other hand, hypertriglyceridemia is conventionally considered a weaker risk factor for atherosclerosis compared to raised LDL-cholesterol [30]. Consequently, the accumulative impact of smoking, together with dyslipidemia, has been proven to be a nasty risk factor for coronary artery events [31,32] and stroke. Separating those risk factors individually as predictors of coronary events is not always easy; however, to identify the exact pattern of acute coronary artery disease and its related risk factors, we conducted our analysis in such a manner. We succeeded in identifying smoking and high triglycerides as the two main predictors of STEMI in Kosovo. While smoking would easily be accepted as an important risk for STEMI, hypertriglyceridemia needs further studies and comparisons with internationally published data. 

Clinical Implications: The findings of this large cohort of Kosovo patients with acute MI reveals that there is a serious need for better atherosclerosis risk factor control with its well-tested beneficial impact on reducing the incidence of acute MI with its short- and long-term complications, as has previously been shown [19,33,34,35,36]. Such control needs a multidisciplinary strategy between health professionals and politicians, and it should also have national implications irrespective of the population size, at least with a serious revolution against smoking, dyslipidemia, and various hypertension-related risks. It would be of interest to analyze the pattern of risk factors contributing to acute strokes in Kosovo, the results of which should strengthen the atherosclerosis disease prevention strategy. 

Limitations: The data collected in this study were retrospective but fulfilled all required and recognized criteria used before in other national registries. We would have liked to have more biomarkers to enrich the search for predictors, but those were limited to conventionally analyzed ones used in daily practice because of the retrospective nature of this study. Despite that, we succeeded in collecting data from all patients admitted with acute MI without a single case missed. The exact interpretation of the link between hypertriglyceridemia and STEMI needs further detailed studies.

## 5. Conclusions

In Kosovo, the majority of patients with acute MI presented with STEMI, and they were mostly males and were more likely to have diabetes, arterial hypertension, and a positive family history of CAD, but they smoked less compared to the patients with NSTEMI. Smoking and hypertriglyceridemia proved to be the strongest predictors of acute STEMI in Kosovo, thus highlighting the urgent need for better tailored atherosclerosis risk control and education strategies. 

## Figures and Tables

**Table 1:** Clinical and biochemical data of patients with acute myocardial infarction.

Variable	All Included Patients	Patients with NSTEMI	Patients with STEMI	*p*-Value
	(*n* = 7353)	(*n* = 2987)	(*n* = 4366)	
Age (years)	63 ± 12	63 ± 11	64 ± 12	0.077
Female (%)	28.7	31.7	26.6	˂0.001
Smoking (%)	51.7	48.3	54	˂0.001
Diabetes (%)	35.3	37.8	33.6	˂0.001
Hypercholesterolemia (%)	40.1	39.4	40.1	0.099
Arterial hypertension (%)	65.6	69.6	63	<0.001
Family history of CAD (%)	38.1	39.9	36.9	0.009
Atrial fibrillation (%)	4.4	3.9	5.0	0.036
Left bundle branch block (%)	3.7	2.6	5.3	<0.001
Cardiogenic shock (%)	3.7	2.1	4.9	<0.001
Fasting glucose (mmol/L)	9.5 ± 5.5	9.2 ± 5	9.8 ± 6	<0.001
Total cholesterol (mmol/L)	4.8 ± 1.6	4.8 ± 1.6	4.5 ± 1.5	0.939
Triglycerides (mmol/L)	1.9 ± 1.3	2.0 ± 1.3	1.88 ± 1.2	0.001
Creatinine (μmol/L)	118 ± 74	118 ± 74	117 ± 73	0.632
Urea (mg/dL)	9.2 ± 6	9.2 ± 6	9.1 ± 6	0.652
Hemoglobin (g/dL)	13.7 ± 3.2	13.7 ± 3.4	13.7 ± 3	0.942
Heart rate at admission (beats/min)	82.6 ± 23	82.5 ± 26	82.6 ± 20	0.822
Left ventricular ejection fraction (%)	50.6 ± 9	51.6 ± 9	49.8 ± 9	<0.001

Legend: STEMI: ST-segment elevation myocardial infarction; NSTEMI: non-ST-segment elevation myocardial infarction; CAD: coronary artery disease.

**Table 2:** Invasive treatment of patients with acute myocardial infarction.

Variable	All Included Patients	Patients with NSTEMI	Patients with STEMI	*p*-Value
	(*n* = 7353)	(*n* = 2987)	(*n* = 4366)	
Coronary Angiography (%)	67.3	66.8	67.8	0.396
Primary Percutaneous Intervention (%)	50.1	43.6	55.2	<0.001

Legend: STEMI: ST-segment elevation myocardial infarction; NSTEMI: non-ST-segment elevation myocardial infarction.

**Table 3:** Associates of STEMI in patients with acute MI.

	Univariate Associates			Multivariate Associates		
Variable	OR	(CI 95%)	*p*-Value	OR	(CI 95%)	*p*-Value
Age	0.993	(0.989–1.997)	0.001			
Female gender	0.782	(0.706–0.876)	<0.001			
Smoking	0.796	(0.725–0.874)	<0.001	0.783	(0.685–0.895)	<0.001
Diabetes	1.202	(1.090–1.324)	<0.001			
Arterial hypertension	1.347	(1.220–1.488)	<0.001			
Family anamnesis for CAD	1.135	(1.032–1.249)	0.009			
Triglycerides	0.943	(0.901–0.987)	0.012	0.946	(0.902–0.991)	0.020

Legend: STEMI: ST-segment elevation myocardial infarction; CAD: coronary artery disease.

**Table 4:** The prevalence of risk factors in Kosovo compared to Finland and Greece in patients admitted with acute MI.

Variable	Kosovo	Finland		Kosovo	Greece	
	(*n* = 7353)	(*n* = 1813)	*p*-Value	(*n* = 7353)	(*n* = 1185)	*p*-Value
Age (years) Female (%)	63 ± 12 28.7	71 ± 13 34.6	<0.000 <0.001	63 ± 12 28.7	67 ± 13 26.2	<0.001 <0.001
Smoking (%) Diabetes (%)	51.7 35.3	20.4 24.8	<0.001 <0.001	51.7 35.3	41.4 27.6	<0.001 <0.001
Arterial hypertension (%) STEMI (%)	65.6 59.4	53.4 34.7	<0.001 <0.001	65.6 59.4	52.6 35.3	<0.001 <0.001
NSTEMI (%)	40.6	65.3	<0.001	40.6	64.7	<0.001

Legend: STEMI: ST-segment elevation myocardial infarction; NSTEMI: non-ST-segment elevation myocardial infarction.

## Data Availability

Data are available upon request due to restrictions (privacy, legal, or ethical reasons).
